# Supplementary material for: Viral and host factors associated with SARS-CoV-2 disease severity in Georgia, USA
Source: medRxiv. 2023 Oct 25:2023.10.25.23297530. Preprint. [Version 1] doi: 10.1101/2023.10.25.23297530 (PMC10635197; doi:10.1101/2023.10.25.23297530)
Supplement: Supplement 1 [file NIHPP2023.10.25.23297530v1-supplement-1.pdf]

medRxiv preprint doi: https://doi.org/10.1101/2023.10.25.23297530; this version posted October 25, 2023. The copyright holder for this preprint (which was not certified by peer review) is the author/funder, who has granted medRxiv a license to display the preprint in perpetuity.
It is made available under a CC-BY 4.0 International license .

585 # SUPPLEMENTARY TABLES

Table S1. Extended COVID Symptoms and Underlying Conditions by Vaccination Status

Variable	Overall, N = 1,957[1]	Unvaccinated, N = 1,024[1]	Vaccinated, N = 933[1]	p [2]
**COVID Symptoms**				
Fever	720 (47%)	430 (52%)	290 (42%)	<0.001
(Missing)	435	197	238	
Chills	537 (35%)	314 (38%)	223 (32%)	0.02
(Missing)	435	198	237	
Rigors	29 (2.0%)	17 (2.1%)	12 (1.8%)	0.67
(Missing)	491	221	270	
Myalgia	501 (34%)	279 (35%)	222 (33%)	0.59
(Missing)	484	218	266	
Headache	521 (35%)	299 (37%)	222 (33%)	0.12
(Missing)	476	215	261	
Sore Throat	286 (20%)	138 (17%)	148 (22%)	0.02
(Missing)	496	228	268	
Nausea / Vomiting	451 (30%)	282 (35%)	169 (25%)	<0.001
(Missing)	455	209	246	
Diarrhea	369 (25%)	212 (26%)	157 (23%)	0.13
(Missing)	470	220	250	
Fatigue	630 (41%)	337 (41%)	293 (41%)	0.81
(Missing)	418	195	223	
Runny Nose or Nasal Congestion	519 (35%)	217 (27%)	302 (45%)	<0.001
(Missing)	473	215	258	
Cough	1,087 (70%)	576 (69%)	511 (72%)	0.37
(Missing)	414	195	219	
Shortness of Breath or Difficulty Breathing	690 (44%)	408 (49%)	282 (39%)	<0.001
(Missing)	401	188	213	
Loss of Taste	264 (18%)	151 (19%)	113 (17%)	0.33
(Missing)	505	233	272	
**Underlying Conditions**				
Pregnant	69 (3.6%)	59 (6.0%)	10 (1.1%)	<0.001
(Missing)	66	42	24	
Chronic Lung Disease	387 (21%)	193 (20%)	194 (22%)	0.33
(Missing)	134	75	59	
Hypertension	874 (47%)	377 (39%)	497 (56%)	<0.001
(Missing)	95	50	45	
Overweight	584 (32%)	315 (33%)	269 (32%)	0.50
(Missing)	150	70	80	
Cardiovascular Disease	534 (29%)	217 (23%)	317 (36%)	<0.001
(Missing)	126	65	61	
Diabetes	426 (23%)	180 (19%)	246 (28%)	<0.001
(Missing)	101	53	48	

medRxiv preprint doi: https://doi.org/10.1101/2023.10.25.23297530; this version posted October 25, 2023. The copyright holder for this preprint (which was not certified by peer review) is the author/funder, who has granted medRxiv a license to display the preprint in perpetuity.
It is made available under a CC-BY 4.0 International license .

Renal Disease	316 (17%)	106 (11%)	210 (24%)	<0.001
(Missing)	132	69	63	
Liver Disease	83 (4.5%)	37 (3.8%)	46 (5.2%)	0.16
(Missing)	107	59	48	
Autoimmune Disease	101 (5.5%)	42 (4.4%)	59 (6.8%)	0.03
(Missing)	135	69	66	
Immunocompromised[3]	269 (15%)	89 (9.3%)	180 (21%)	<0.001
(Missing)	137	69	68	
Systemic Immunosuppressive Therapy or Medications	293 (16%)	117 (12%)	176 (20%)	<0.001
(Missing)	115	59	56	

[1] n (%) or median (Interquartile Range [IQR])
[2] Pearson's Chi-squared test or Fisher's exact test for categorical variables and Wilcoxon rank-sum test for continuous variables
[3] Immunocompromised (e.g., HIV infection, active cancer, solid organ transplant, hematopoietic stem cell transplant)

Table S2. Circulation of VOC/VOI in Georgia, USA as measured by available sequences on GISAID

VOC/VOI	Period variant circulated in GA[1]	Date range of sequences obtained from GISAID	Sequences available on GISAID	Total sequences available from May 2021 to May 2022 (%)
**Alpha**	12/10/2020-08/26/2021	05/01/2021-08/26/2021	1,361	2.66
**Beta**	02/01/2021-05/25/2021	05/01/2021-05/25/2021	16	0.03
**Delta**	4/29/2021-03/10/2022	05/01/2021-03/10/2022	28,320	55.5
**Gamma**	03/05/2021-09/24/2021	05/01/2021-09/24/2021	185	0.36
**Lamba**	02/07/2021-08/05/2021	05/01/2021-08/05/2021	18	0.04
**Mu**	04/23/2021-09/14/2021	05/01/2021-09/14/2021	142	0.28
**Omicron**	11/30/2021-4/18/2023	11/30/2021-04/18/2023	20,759	40.6
		**Total VOC/VOI**	**50,801**	**99.46**
**Total sequences available on GISAID from May 2021 to May 2022 from the state of Georgia**			**51,075**	**---**

[1]Period of circulation based on sequences available on GISAID including Emory Healthcare samples.

medRxiv preprint doi: https://doi.org/10.1101/2023.10.25.23297530; this version posted October 25, 2023. The copyright holder for this preprint (which was not certified by peer review) is the author/funder, who has granted medRxiv a license to display the preprint in perpetuity.
It is made available under a CC-BY 4.0 International license .

Table S3. Unadjusted Multinomial Logistic Regression Models Testing the Association between Demographics, Underlying Health Conditions, and COVID-Related Characteristics with Disease Severity

	Moderate[1]			Severe[1]			Death[1]		
**Variable**	**OR[2]**	**95% CI[2]**	**p**	**OR[2]**	**95% CI[2]**	**p**	**OR[2]**	**95% CI[2]**	**p**
**Unadjusted Models**									
**Age**	**1.04**	**1.03, 1.05**	**<0.001**	**1.05**	**1.04, 1.05**	**<0.001**	**1.07**	**1.05, 1.09**	**<0.001**
**Male[3]**	**1.40**	**1.13, 1.74**	**<0.01**	1.30	0.95, 1.78	0.10	1.26	0.72, 2.22	0.40
White[4]	0.86	0.67, 1.10	0.20	1.27	0.91, 1.77	0.20	1.04	0.57, 1.91	0.90
**Chronic Lung Disease**	**2.07**	**1.59, 2.68**	**<0.001**	**1.95**	**1.35, 2.81**	**<0.001**	**2.65**	**1.44, 4.86**	**<0.01**
**Hypertension**	**2.94**	**2.35, 3.68**	**<0.001**	**4.19**	**2.98, 5.90**	**<0.001**	**11.4**	**5.09, 25.6**	**<0.001**
**Overweight**	**1.45**	**1.15, 1.83**	**<0.01**	**1.58**	**1.14, 2.19**	**0.01**	**2.02**	**1.13, 3.61**	**0.02**
**Cardiovascular Disease**	**2.89**	**2.28, 3.67**	**<0.001**	**3.46**	**2.48, 4.81**	**<0.001**	**4.80**	**2.70, 8.54**	**<0.001**
**Diabetes**	**2.04**	**1.58, 2.63**	**<0.001**	**3.21**	**2.29, 4.50**	**<0.001**	**4.35**	**2.43, 7.80**	**<0.001**
**Renal Disease**	**3.88**	**2.90, 5.19**	**<0.001**	**3.86**	**2.62, 5.67**	**<0.001**	**9.86**	**5.46, 17.8**	**<0.001**
**Liver Disease**	1.47	0.87, 2.50	0.20	**2.36**	**1.25, 4.45**	**<0.01**	**3.08**	**1.16, 8.21**	**0.02**
**Autoimmune Disease**	**1.60**	**1.01, 2.56**	**0.046**	**1.58**	**0.82, 3.03**	**0.20**	**2.45**	**0.93, 6.47**	**0.07**
**Immunocompromised**	**2.65**	**1.97, 3.58**	**<0.001**	**2.44**	**1.61, 3.69**	**<0.001**	**3.69**	**1.93, 7.09**	**<0.001**
**Systemic Immunosuppressive Therapy/Meds.**	**3.49**	**2.61, 4.67**	**<0.001**	**3.24**	**2.18, 4.81**	**<0.001**	**3.48**	**1.79, 6.78**	**<0.001**
Vaccination Status									
Unvaccinated	Ref	—		Ref	—		Ref	—	
**Vaccinated[5]**	**0.80**	**0.64, 1.01**	**0.07**	0.86	0.61, 1.21	0.40	1.00	0.54, 1.84	>0.90
**Vaccinated & boosted[5]**	**2.28**	**1.52, 3.43**	**<0.001**	**3.24**	**1.95, 5.40**	**<0.001**	**3.69**	**1.58, 8.59**	**<0.01**
Days Since Most Recent Vaccination/Booster									
Unvaccinated	—	—		—	—		—	—	
Within past 90 Days[5]	1.34	0.86, 2.08	0.20	1.68	0.93, 3.03	0.09	2.81	1.17, 6.79	0.02
91 - 180 Days Ago[5]	0.98	0.74, 1.29	0.90	0.92	0.60, 1.40	0.70	1.14	0.55, 2.37	0.70
181 - 270 Days Ago[5]	0.72	0.51, 1.01	0.06	0.74	0.45, 1.24	0.30	0.71	0.27, 1.89	0.50
More than 270 Days Ago[5]	1.18	0.72, 1.94	0.50	2.49	1.43, 4.36	<0.01	1.91	0.64, 5.72	0.20
Lineage									
Delta	Ref	—		Ref	—		Ref	—	
**Omicron[6]**	**1.51**	**1.09, 2.07**	**0.01**	**2.25**	**1.46, 3.48**	**<0.001**	**4.55**	**2.25, 9.20**	**<0.001**

[1]" Mild" is the Reference Category
[2] OR = Odds Ratio, CI = Confidence Interval, Ref = Reference Level
[3] Compared to "Female"
[4] Compared to "Black"
[5] Compared to "Unvaccinated individuals"
[6] Compared to "Delta"

medRxiv preprint doi: https://doi.org/10.1101/2023.10.25.23297530; this version posted October 25, 2023. The copyright holder for this preprint (which was not certified by peer review) is the author/funder, who has granted medRxiv a license to display the preprint in perpetuity.
It is made available under a CC-BY 4.0 International license .

SUPPLEMENTARY FIGURES

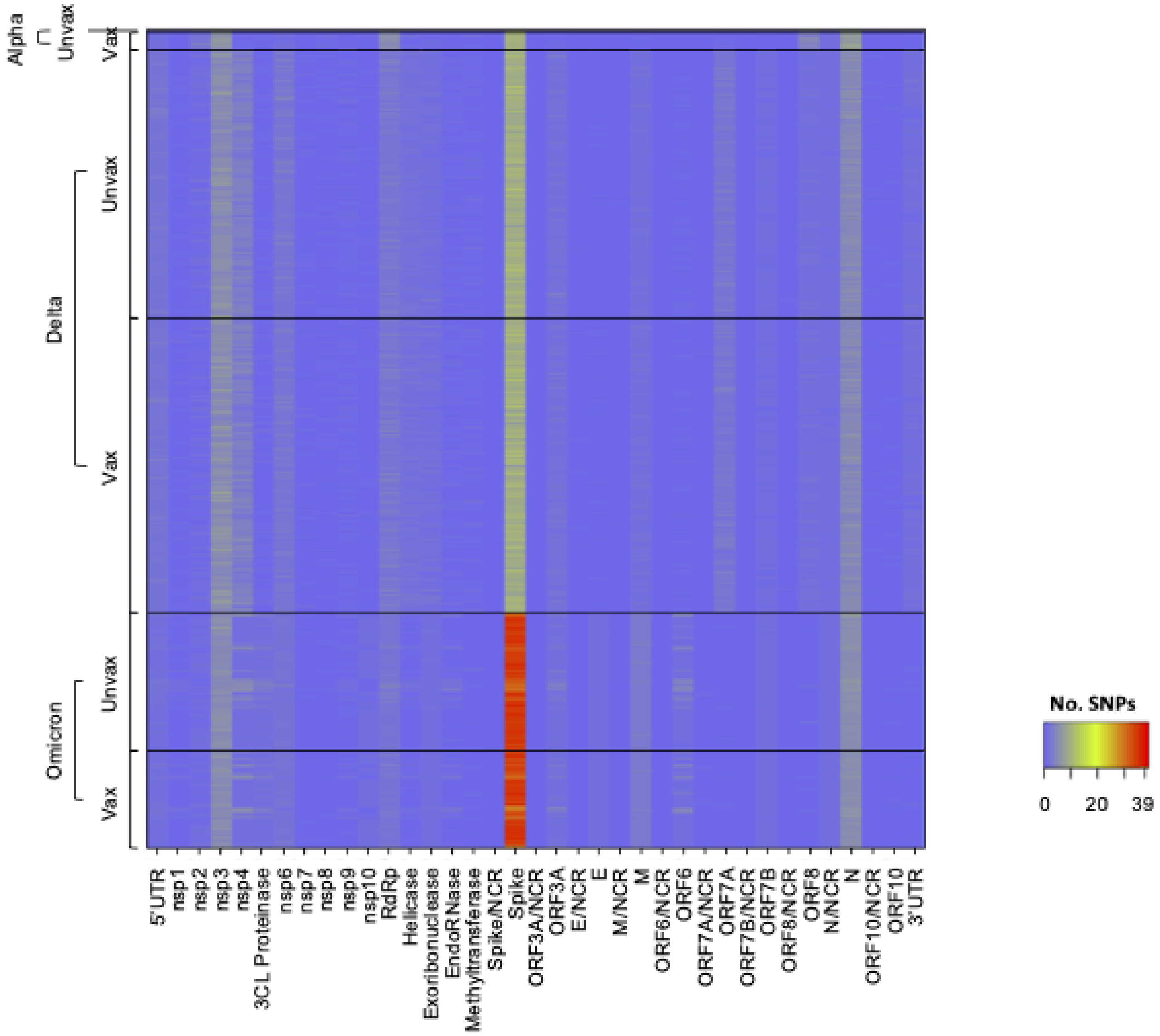

Figure S1. Mutations across SARS-CoV-2 genes. Alpha, delta, and omicron sequences were aligned to Wuhan/Hu-1/2019. For each variant, samples from vaccinated (“vax”) and unvaccinated ("unvax") individuals are labeled. Each square in the heatmap represents the number of single nucleotide polymorphisms (SNPs) in each gene, labeled on the x-axis. Each row represents one sample.

medRxiv preprint doi: https://doi.org/10.1101/2023.10.25.23297530; this version posted October 25, 2023. The copyright holder for this preprint (which was not certified by peer review) is the author/funder, who has granted medRxiv a license to display the preprint in perpetuity.
It is made available under a CC-BY 4.0 International license .

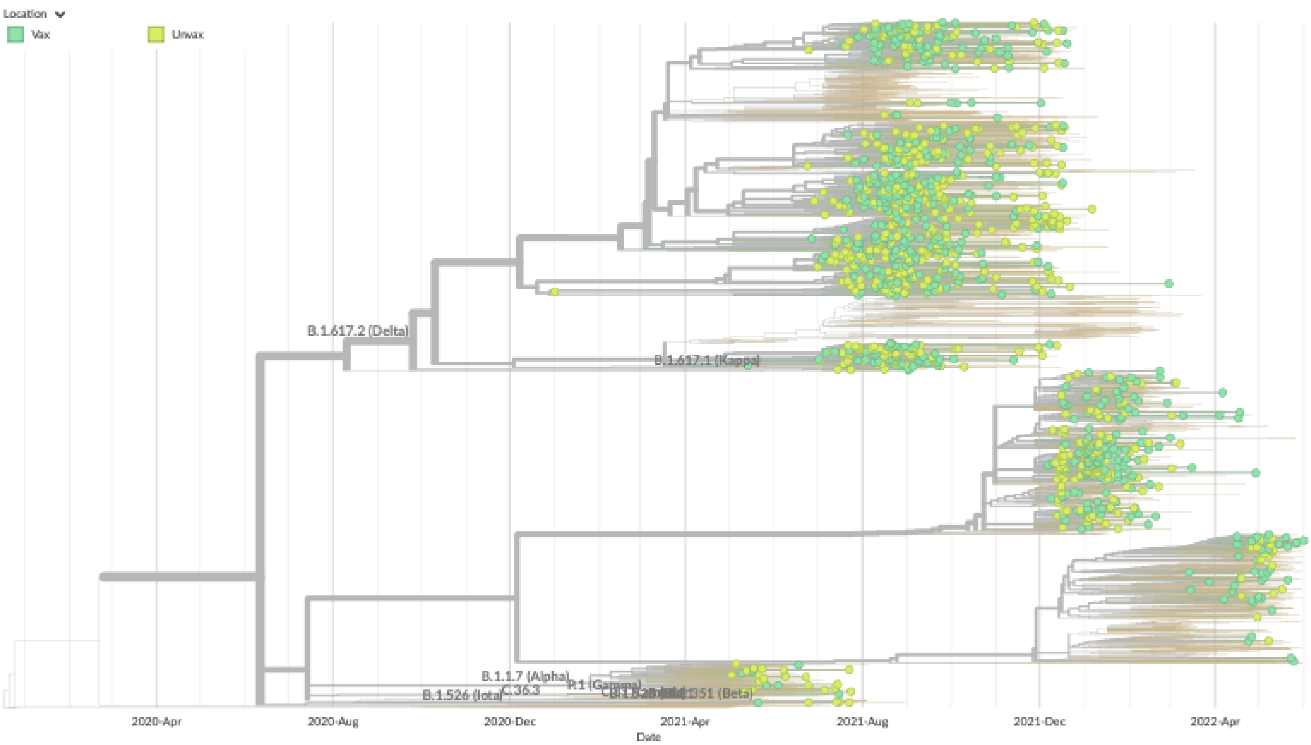

Figure S2. Phylogenetic analysis does not reveal differences in SARS-CoV-2 sequences from vaccinated and unvaccinated individuals. Maximum likelihood tree containing sequences from vaccinated (green) and unvaccinated (yellow) individuals in the context of 2000 global sequences from GISAID (orange) selected by a custom Nextstrain subsampling scheme and rooted to NC_045512.
